# Giving oocytes to women in need: permitted, forbidden, or pressured? A commentary

**DOI:** 10.1186/2045-4015-1-16

**Published:** 2012-04-18

**Authors:** Françoise Shenfield

**Affiliations:** 1 Reproductive Medicine Unit, University College London Hospitals, Euston Road, London NW1 3BU, UK

**Keywords:** cross border reproductive care, egg donation, egg sale, justice, solidarity

## Abstract

There can be no starker contrast in egg donation than between Austria, where it is forbidden, and Israel, where it is encouraged to the extent that some Israeli women go abroad in order to obtain these precious reproductive cells. There are grave ethical issues in some of the transactions involved, which may fall out with the definition of a gift. The ESHRE taskforce on cross-border reproductive care is keen to promote standards in this field, including the ethical standard of avoiding coercion.

This is a commentary on http://www.ijhpr.org/content/1/1/15/

## Commentary

The paper by Shalev and Werner-Felmayer [1] highlights the different approaches in assisted reproduction (ART) in Israel and Austria, and focuses on some of the ethical problems encountered when egg donor recruitment is performed on a purely market basis, treating eggs as a commodity, and not as a precious cell given by generous women to help others.

Interestingly, the call for "harmonization" in legislation comes from these two very different countries--one where assisted reproductive law is restrictive, and where there is little evidence that it is not adhered to, Austria; and the other, Israel, where the law is broad and favors a pro-natalist policy, but where a substantial minority of IVF specialists (22.5%) claimed they did not follow existing national guidelines and "exercise full discretion in offering treatment to patients" [2]. It is not unusual to hear calls for "harmonization" of laws when there are major differences in Europe, but this is not within the remit of the EU, which leaves a fairly wide "margin of appreciation" to different countries in health matters. The only existing unifying document is the Tissue Directive concerning standards for the keeping of human "tissues", which includes gametes [3].

This article, however, concentrates on the differences in gamete donation, particularly oocyte donation, and they cannot be starker, as it is actually forbidden in Austria. There, the situation is rendered more complex as physicians also have a responsibility to the gamete donors.

As fertility specialists, we must ensure that they are not put at disproportionate risk of medical complications, or abused when they sell rather than donate eggs. Again this entails professional responsibility, and this is why ESHRE (European Society of Human Reproduction and Embryology), the European Society representing over 5000 fertility specialists as well as nurses, embryologists, and psychologists, created a special Taskforce three years ago on cross-border reproductive care http://www.ESHRE.eu.

We already know some of the facts: Cross-border reproductive care refers to a widespread phenomenon where infertile patients or collaborators (such as egg donors or potential surrogates) cross borders in order to obtain or provide reproductive treatment outside their home country. The reasons for traveling vary between countries, but the most common reason is law evasion when the technique is either forbidden per se or when a particular group is excluded from treatment. There may be other access limitations at home such as long waiting lists; other reasons are better quality of care and cheaper treatment.

Contrary to the conclusion of the authors in this paper, we do not think it feasible to have any kind of European harmonization of ART legislation. The EU indeed leaves a wide margin of approval to country members in this domain, and the only binding regulatory piece up until now is the EU Tissue Directive, which applies to the taking and keeping (storage) of tissues and gametes (sperm and oocytes included), with the necessary screening of donors and the type of laboratories where they are kept. The "ESHRE Good Practice Guide to Cross Border Reproductive Care" was published in *Human Reproduction *[4].

It is up to the professionals to put their house in order, and thus we wrote this "guide". We are aware of the limitations of "Guidance" compared with a top-heavy system of certification or accreditation. But this must be seen as a first step in a European scene that more and more demands control of standards to ensure the safety of patients, gamete donors, and surrogates, as well as that of future offspring. Our guidance also concerns inter-professional communication, which is available on ESHRE's website, and several statutory authorities, from the British HFEA to the Canadian and Australian equivalents, as well as national fertility societies (Belgian, British, Danish, Portuguese, the Italian Register, and the Spanish Association of Clinical Embryologists for instance), and the European Patient Fertility Association, have links to this guide on their website.

This guide is for centers and physicians treating foreign patients, and may also help regulators and policy makers create a framework to enable centers to abide by these rules. Although in principle the care of foreign and local patients should be the same, and fit the best possible standards, there is evidence that this is not always the case.

We focus on several principles: equity, safety, efficiency, effectiveness (including evidence-based care), timeliness, and patient centeredness for patients, gamete donors, surrogates, and professionals.

In the case of gamete donation, which is at the core of the paper published here, we advise that it is essential to follow the recommendations of the EU Tissues Directive, with special regard to the screening process and the non-commercialization conditions. The latter condition, if followed, would therefore avoid the situation described by Shalev and Werner-Felmayer concerning the commercialization of egg donation, whilst arguably the lack of proper consent is at least ethically as damaging to the whole field of ART.

We conclude that it is essential first, to establish national registers of gamete donors, and second, for centers to participate in the collection of national or international data. Transparency is one of the keys to confirming the trust that should prevail in gamete donation treatment, when neither the patients nor the profession wishes to abuse other women to obtain a much desired child in the great majority of cases. But when such abuse occurs, even if rare in comparison to the number of cycles performed, trust towards the profession is damaged, and thus specialists in the field have to show that they obtained oocytes from consenting women, and without disproportionate compensation.

ESHRE plans further studies on oocyte donors in Europe, and a more detailed good practice in this field will probably ensue.

## Author's information

Françoise Shenfield is a fertility specialist with more than 25 years of experience at the University College London Hospitals. She is also qualified in medical law and ethics, is a member of the European Society of Human Reproduction and Embryology's Taskforce for Ethics and Law, and is a co-chair of the International Federation of Gynecology and Obstetrics' ethics committee.

## Competing interests

The author declares that they have no competing interests.
